# Osteopontin, a bridge links osteoarthritis and osteoporosis

**DOI:** 10.3389/fendo.2022.1012508

**Published:** 2022-10-28

**Authors:** Rui-Jun Bai, Yu-Sheng Li, Fang-Jie Zhang

**Affiliations:** ^1^ Department of Orthopaedics, Xiangya Hospital, Central South University, Changsha, Hunan, China; ^2^ National Clinical Research Center for Geriatric Disorders, Xiangya Hospital, Changsha, Hunan, China; ^3^ Department of Emergency Medicine, Xiangya Hospital, Central South University, Changsha, Hunan, China

**Keywords:** bone metabolism, inflammation, osteoarthritis, osteoporosis, osteopontin

## Abstract

Osteoarthritis (OA) is the most prevalent joint disease characterized by degradation of articular cartilage, inflammation, and changes in periarticular and subchondral bone of joints. Osteoporosis (OP) is another systemic skeletal disease characterized by low bone mass and bone mineral density (BMD) accompanied by microarchitectural deterioration in bone tissue and increased bone fragility and fracture risk. Both OA and OP are mainly affected on the elderly people. Recent studies have shown that osteopontin (OPN) plays a vital role in bone metabolism and homeostasis. OPN involves these biological activities through participating in the proliferation, migration, differentiation, and adhesion of several bone-related cells, including chondrocytes, synoviocytes, osteoclasts, osteoblasts, and marrow mesenchymal stem cells (MSCs). OPN has been demonstrated to be closely related to the occurrence and development of many bone-related diseases, such as OA and OP. This review summarizes the role of OPN in regulating inflammation activity and bone metabolism in OA and OP. Furthermore, some drugs that targeted OPN to treat OA and OP are also summarized in the review. However, the complex mechanism of OPN in regulating OA and OP is not fully elucidated, which drives us to explore the depth effect of OPN on these two bone diseases.

## Introduction

Osteoarthritis (OA), the most common aging-related joint pathology is a degenerative disease affecting all the structures of the joints. OA is mainly characterized by articular cartilage destruction along with changes occurring in other joint components including bone, menisci, synovium, ligaments, capsule, and muscles (1). Worldwide estimates that 9.6% of men and 18.0% of women aged over 60 years have symptomatic OA (2). Radiographic evidence of OA occurs in the majority of people by 65 years of age and in about 80% of those aged over 75 years (3).

Osteoporosis (OP) is defined as a systemic skeletal disease characterized by low bone mass and micro-architectural deterioration of bone tissue with a consequent increase in bone fragility and susceptibility to fracture (4). OP is a major risk factor for fractures of the hip, vertebrae, and distal forearm. It is thought of as a disease of old age which is present in 15% of those 50–59 years of age, but these figures increase quickly to 70% of those over 80 years of age (2). The relationship between OA and OP is complex and controversial. Kim et al. (5) conducted a meta-analysis and finally found the frequency of OP overall in men and women with OA was no different. However, according to the site of bone mineral density measurement, there was a higher prevalence of OP in the lumbar spine in both men and women compared to the matched controls. A cross-sectional study which was aimed to reveal the relationship between radiographic features of OA and bone mineral density (BMD) found that hand osteophytes and sclerosis exhibited a positive relationship with the BMDs of the lumbar spine and femoral neck while the knee and hand joint space narrowing presented a negative tendency to the BMD of the lumbar spine and femoral neck (6). Kasher et al. (7) confirmed that a higher hand OA score was significantly negatively correlated with arm and hand BMD measurements in males and females accompanied by a higher prevalence of wrist fracture, but the knee OA affection was positively associated with the elevated hip, spine, and total body BMD levels. A previous study also found the relationship between OA and OP was sexually different for the reason that femur neck and lumbar BMD and OA showed a positive tendency in women while BMD of the lumbar and pelvis in men was negatively correlated with OA (8). Hence, illuminating the relationship between OA and OP may uncover the relationship between OA and OP as well as the molecular or pathological factors that influence them that could help treat these diseases.

Some factors have been found to affect the progress of both OA and OP, including sex hormones, ethnicity, age, nutritional factors, genetic factors and physical activity. Aging is a predictor of radiographic OA, bone loss, development of OP, and fracture (2). Recently, a few studies found that the expression of OPN mRNA isolated from human OA cartilage was enhanced as compared with normal cartilage. OPN was shown to be upregulated in human OA chondrocytes (9). OPN in plasma, synovial fluid and articular cartilage is associated with progressive joint damage and is likely to be a useful biomarker for determining disease severity and progression in knee OA (10, 11). Additionally, high serum OPN level was also a significant risk factor causing menopausal OP and serum OPN levels could be used as a biomarker for the early diagnosis of OP in postmenopausal women (12). Thus, OPN may be involved in the molecular pathogenesis of OA and OP, it may play a role as a bridge between OA and OP. Herein, we summarize current understandings of the molecular mechanism of OPN in OA and OP, focusing on recent results that have examined the role of OPN between OA and OP.

## The structure and function of OPN

OPN is a 44-75 KD multifunctional phosphoprotein secreted by many cell types such as osteoclasts, chondrocytes, synoviocytes, macrophages, lymphocytes, epithelial cells and vascular smooth muscle cells (SMC) and is present in the extracellular matrix of mineralized tissues and extra-cellular fluids, at sites of inflammation (13–15). It is encoded by the SPP1 gene and maps as a tandem array to the long arm of chromosome-4 (16), producing splicing variants of mRNA, full-length OPN and spliceosomal OPN (missing exons 4 or 5) (17, 18). The full-length OPN is cleaved by thrombin to form the thrombin-cleaved OPN (19). Both the native OPN and cleaved OPN could interact with integrin and play an important role in regulating inflammation, biomineralization, bone remodeling, immune functions, chemotaxis, and cell apoptosis (19, 20). Gene structure and chromosomal location identify OPN as a member of the small integrin-binding ligand N-linked glycoprotein (SIBLING) family. This protein also known as early T cell activation gene-1 (Eta-1) is abundant in bone, where it mediates important cell-matrix and cell-cell interactions (21). OPN expression is one of the important events involved in cartilage-to-bone transitions in fracture repair during the period of chondrocyte maturation (22, 23). OPN facilitates the attachment of osteoclasts to the bone matrix *via* interaction with cell surface αvβ3 integrin and CD44 (24, 25). OPN interacts with receptors such as integrin and CD44 to regulate physiological and pathological processes of proliferation, differentiation, inflammation, metabolism and tumor metastasis in chondrocytes, osteoblasts and osteoclasts (26–28). Previous studies have shown that OPN is involved in the occurrence, repair and maintenance of cartilage and subchondral bone metabolic homeostasis, suggesting that OPN is an important regulator of OA and OP and plays an important role in chondrocyte and osteocyte metabolism by regulating the extracellular matrix components of articular cartilage subchondral bone components (10, 29, 30).

OPN is widely distributed in many cells and tissues such as chondrocytes, plasma, synovial, osteoblasts and osteoclasts and plays regulatory roles in many diseases. Clinical studies have shown that OPN is involved in bone strength and remodeling, suggesting that serum OPN is positively correlated with the severity of OP and could be targeted as a biomarker for early diagnosis of postmenopausal osteoporosis (31, 32). Further, OPN is related to bone turnover and bone mineral density (BMD) and influences morphological formation and reconstruction (33–35). The high serum OPN level could result in a low BMD and OP in postmenopausal women (34, 35), moreover, the high level of OPN is associated with osteoporotic fractures in postmenopausal women, particularly at the lumbar spine (32). In addition, researchers have examined the relation between obesity and OP, and suggested obesity could lead to a large number of adipocytes and adipose tissue which may be greatly related to OP (36–38). Dai et al. (39) found that OPN secreted by the macrophages in epididymal white adipose tissue regulated the bone metabolism in high-fat diet (HFD)-induced obesity. The OPN selectively circulated to the bone marrow and promoted the degradation of the bone matrix by activating osteoclasts, both surgical removal of epididymal white adipose tissue and local injection of OPN-neutralizing antibodies or drugs aimed to deplete macrophages could ameliorate HFD-induced bone loss in mice. There is also evidence indicating that OPN is one of the most overexpression genes in the adipose tissue-derived from obese patients (40).

OPN is a critical intrinsic regulator that plays an important role in the pathogenesis and progress of OA. The plasma and synovial fluid level of OPN in OA patients are higher than in healthy adults, suggesting that OPN may be correlated with the severity of joint lesions in OA (11). Min et al. (41) investigated the serum of 249 people and found serum OPN was significantly increased in OA patients compared with control group, furthermore, they found there existed a gender difference in the concentration of OPN between the OA group and the control group. The gender difference in OPN expression was mainly presented that serum OPN level in the control group was lower in OA patients with the exact level of 2539.9 (pg/ml) and 5538.1 (pg/ml) in males, while the results of serum OPN level in the control group compared to OA patients were 1632.0 (pg/ml) and 4545.8 (pg/ml) in female. In addition, other researchers also found higher OPN mRNA and protein expression in synovial fluid of OA patients is closely related to the occurrence and development of OA (42), while OPN gene polymorphism could decrease the risk of OA (43). Recently, studies also show the expression of OPN is greatly increased in both the superficial zone and deep zone of articular cartilage of OA patients (9, 44). Animal experiments show that increased expression of OPN accelerates the turnover and remodeling of OA subchondral bone, promotes vessels formation in subchondral bone, mediates articular cartilage degeneration induced by subchondral bone metabolism, and accelerates the progression of OA (45). Some contradictory findings show OPN has a therapeutic value and mechanism in OA treatment. The mRNA and protein expression of OPN, CD44 is upregulated in OA chondrocytes, moreover, the upregulated OPN could delay chondrocyte degeneration and reduce cartilage matrix component loss by binding to CD44 and integrin *via* OPN/CD44/PI3K signaling pathway (46). CD44 is a cell surface protein that interacts with a variety of extracellular matrix (ECM) components whose principal ligands mainly include hyaluronan (HA) and OPN (47). The CD44 variants containing the exons v6 and v7 bind to the N- and C- terminal portions of OPN in an arginine-glycine-aspartic independent manner and further regulate the effect of OPN on bone metabolism (48). Zhang et al. (15) indicated that HA could upregulate OPN mRNA expression in OA fibroblast-like synoviocytes, and the high expression of OPN mRNA in OA may be a result of increased HA level of OA synovitis, finally, alleviating the severity and improving the symptoms of OA. OPN deficiency may enhance the senescence and apoptosis of OA chondrocytes, up-regulated the expression of pro-inflammation, and decrease the expression of COL2A1, finally accelerating the progress of chondrocytes and OA severity (27).

## OPN involves inflammation in OA and OP

OPN is highly expressed in the process of inflammation. Many studies have characterized the function of OPN in inflammation activity, and it plays an important role in the pathogenesis of various inflammatory diseases such as OA. The previous study has shown that OPN is a proinflammatory cytokine that plays an important role in the pathogenesis of arthritis as summarized in **Table 1**. There presents an elevated level of OPN in synovial fluid samples from RA patients, and the increased expression of OPN is correlated with the high level of multiple inflammatory cytokines including tumor necrosis factor-alpha (TNF-α) and interleukin-6(IL-6) (62). Another study also confirms the high protein expression of OPN in synovial fluid derived from advanced OA patients, significant differences and correlations are found among the thrombin-cleaved OPN, synovitis, and cartilage damage indicated by higher Kellgren-Lawrence scores (63). A long-term cohort study has shown the inflammatory mediators mainly including CRP, IL-6 accompanied by OPN significantly increased in arthroplasty patients the day after surgery and returned to and baseline six weeks later. It also found other inflammatory factors IL-1β and IL-8 showed the same up-regulated trend and reached the peak at 5 years post-surgery and returned to normal level at 10 years while the TNF-α level did not show any change preoperative or postoperative (53). The serum sample collected from total joint arthroplasty patients showed OPN and matrix metalloproteinase-9 (MMP-9) was greatly up-regulated, while MMP-9 was known to cleave OPN aimed to degrade Cola2a1 and upregulated by inflammatory markers such as IL-1, IL-6 and CRP (49). These biomarkers of inflammation were correlated with the progress of OA and total joint arthroplasty. The OPN level also significantly increased in synovial fluid samples from symptomatic primary knee osteoarthritis with ultrasound-confirmed joint effusion, moreover, the up-regulated OPN level presented associations between IL-8 and TNF which is responsible for pain, cartilage damage, clinical severity, and progression of OA (54). Wang, et al. (50, 51) also revealed that OPN could regulate the expression of various inflammatory factors including MMP-13, IL-6 and IL-8 which were significantly upregulated in OA tissues and associated with the pathogenesis of OA. Furthermore, many drugs have been devoted to investigating the relationship between OPN and inflammation factors in OA. Isorhamnetin could reduce knee swelling and alleviate cartilage damage in MIA-induced rats. The therapeutic effect was achieved mainly through inhibiting the expression of OPN and C-terminal telopeptide of type II collagen (CTX-II) accompanied by decreased levels of NO, PGE2, iNOS and COX- 2 (52). However, some controversial results found that OPN plays a protective role in OA. Tian et al. (27) compared the mRNA expression level of OPN from human OA chondrocytes and normal chondrocytes, and found the mRNA level of OPN was greatly suppressed in normal chondrocytes. OPN deficiency increased the expressions of Col10a1, IL-1β, TNF-ɑ, MMP-13, and ADAMTS-5 but decreased the expression of Col2a1, finally leading to higher rates of senescence and apoptosis of chondrocytes. Other researchers also found that OPN deficiency led to the induction of MMP-13 in instability-induced and aging-associated OA, which degrades a major component of the cartilage matrix protein type II collagen, indicating OPN plays a pivotal role in the progression of OA (55). Therefore, OPN might be a critical biomarker in the inflammatory activity of OA for either up-regulated OPN level or OPN deficiency could stimulate the secretion of inflammatory factors, damage articular cartilage, and aggravate the severity of OA.

**Table:** Table 1 OPN involves inflammation activity in OA and OP.

Reference	disease	Subjects	cytokines	Main results
Hannah Slovacek, et al. (49)	OA	Blood samples	MMP-9, ADAMTS-4	OPN and ADAMTS-4 inversely fluctuated post-operatively of total joint arthroplasty upregulation of MMP-9 and OPN results in the downregulation of ADAMTS-4.
Jian Tian, et al. (27)	OA	Chondrocytes	IL-1β, TNF-ɑ, MMP-13, ADAMTS-5	OPN deficiency increased the expressions of COL10A1, IL-1β, TNF-ɑ, MMP-13, and ADAMTS-5, but decreased the expression of COL2A1 in chondrocytes, finally accelerated OA progress.
Qiyuan Wang, et al. (50)	OA	Synoviocytes	MMP-13, IL-6, IL-8	OPN upregulates expression of inflammatory factors MMP-13, IL-6 and IL-8 in OA tissues. Inhibiting OPN expression and synoviocyte proliferation is an efficient strategy for OA treatment.
Shenglong Li, et al. (51)	OA	Rat adipose mesenchymal stem cell	IL-1β	IL-1β treatment significantly inhibites the viability and migration of chondrocytes, enhances cell apoptosis andinduced cartilage damage by upregulating IL-1β, OPN, p53 and decreasing the expression of COL1A1, COL2A1, OCN, RUNX2.
Tsai Sen-Wei, et al. (52)	OA	Sprague-Dawley rats	NO, PGE2, iNOS, COX-2	OPN level was inhibited by isorhamnetin accompanied with the down-regulated level of NO, PGE2, iNOS and COX-2 in MIA-induced OA rats.
Jean Cassuto, et al. (53)	OA	Blood samples	CRP, IL-6	Inflammatory mediators CRP, IL-6 increased significantly on day one after surgery vs preoperative value and returned to baseline at 6 week in arthroplasty patients.
María García-Manrique, et al. (54)	OA	Blood samples and synovial fluid	IL-8, TNF	IL-8 in synovial fluid is related to clinical severity and progression in knee osteoarthritis.
Yuichiro Matsui, et al. (55)	OA	C57BL/6 mice	MMP-13	OPN deficiency leads to the induction of MMP-1, which degrades a major component of the cartilage matrix protein type II collagen in mice.
Bao-Ming Tang, et al. (56)	OP	Sprague-Dawley rats	IL-1β, TNF-α, IL-6	Inflammation mediated osteoporosis presented apparently down-regulated in OPN and OCN levels, higher serum IL-1β, TNF-α, IL-6 levels in ovariectomized rats.
Krzysztof Marycz, et al. (57)	OP	MC3T3-E1 cell lines	iNOS, TNF-α, Il-1β	NHAp/IO@miR-21/124 could enhance osteogenesis through increasing osteogenic markers Runx-2, OPN, Coll-1 level, inhibiting the expression of inflammatory markers TNF-α, iNOs or IL-1β in LPS-stimulated cells.
Xiang Gao, et al. (58)	OP	Lewis rats	TNF-α, IL-6	Salvianolate treatment could increase Osterix, OPN, Runx2 level and decrease TNF-α, IL-6 level in serum and IL-1β protein in TNF-α-induced MC3T3-E1 osteoblasts, finally ameliorate osteopenia and improved bone quality.
Wei-Ming Li, et al. (59)	OP	C57BJ/6 L mice	TNF-α, IL-1β, IL-6, CCL-2	Silenceing angiopoietin-like protein2 (ANGPTL2) reduces nuclear factor of activated T cell c1/4 (NFATC1/4) expressions in macrophage colony-stimulating factor -treated cells, decreases Runx2, OPN and Colla1 level and accompanied with down-regulating level pro-inflammatory cytokines such as TNF-α, IL-1β, IL-6 and CCL-2.
Chen Lei, et al. (60)	OP	C57B/L6 mice and osteoclasts	TNF-α, IL-1β, IL-6, and MMP-1	MLN64-knockdown alleviates the severity of osteoporosis, down-regulates specific genes related to osteoclastogenesis including Runx2 and inflammatory factors such as TNF-α, IL-1β, IL-6, and MMP-1.
Yu-Qiong He, et al. (61)	OP	C57/BL6 mice and MC3T3-E1 osteoblast-like cells	IL-6, IL-1β and NO	Monotropein increases the proliferation and activity of ALP, bone matrix mineralization and OPN in osteoblastic MC3T3-E1 cells, decreases the production of IL-6, IL-1β and NO.

Osteoporosis is a common disease in the aging population and multiple studies have shown that inflammation cytokines greatly influence the pathogenesis of OP (64, 65). Researchers have shown that many pro-inflammatory mediators including cytokines such as TNF-α, IL-1, IL-6, and IL-10 up-regulated in OP and participate in the process of bone resorption or bone mineral density (65–67). As a secreted protein, OPN is greatly related to the inflammatory response in OP, which further influences the process of bone remodeling as the **Table 1** shown. The Animal experiment has shown that OPN and osteocalcin (OCN) level declined in inflammation-mediated osteoporosis (IMO) of ovariectomized rats. The IMO rats represented higher serum tartrate-resistant acid phosphatase (TRAP), CTX-I, and pro-inflammatory factors TNF-α, IL-1β, and IL-6 levels, accompanied by decreased femur BMD, bone mineral content (BMC) and distal femur cancellous bone in IMO rats (56). Gao, et al. (58) demonstrated rheumatoid arthritis induced bone loss and bone quality deterioration, with high bone turnover in collagen-induced arthritis (CIA) rats. The CIA rats showed higher levels of IL-6, and TNF-α in serum accompanied by decreased mRNA and protein levels of Osx (Osterix), OPN which is induced by a TNF-α-induced inflammatory medium *in vitro*. Osteoclasts are multinucleated cells essential for bone resorption and play a central role in the development of OP. The osteoclasts intervened by knock-down angiopoietin-like protein2 could promote expressions of pro-inflammatory cytokines including TNF-α, IL-1β, IL-6, and CCL-2, reduce Runx2, OPN, and Colla1 levels, finally improve bone loss and BMD in osteoporosis mice induced by ovariectomy (59). Some studies found many drugs could regulate the OPN level and inflammation response to alleviate the severity of OP. Monotropein significantly inhibited bone mass reduction and improved bone micro-architectures by enhancing bone formation in osteoporotic mice by suppressing the secretion of IL-6 and IL-1β induced by LPS. The experiment *in vitro* also increased the expression and activity of alkaline phosphatase (ALP) and OPN in osteoblasts (61). Other researchers also confirmed that increasing the expression level of OPN and Runx2 could promote bone remodeling and reduce bone loss in OP accompanied by inhibiting the expression of inflammatory markers TNF-α, iNOS, or IL-1β in LPS-stimulated osteoblastic cells (57, 60).

**Figure 1:**
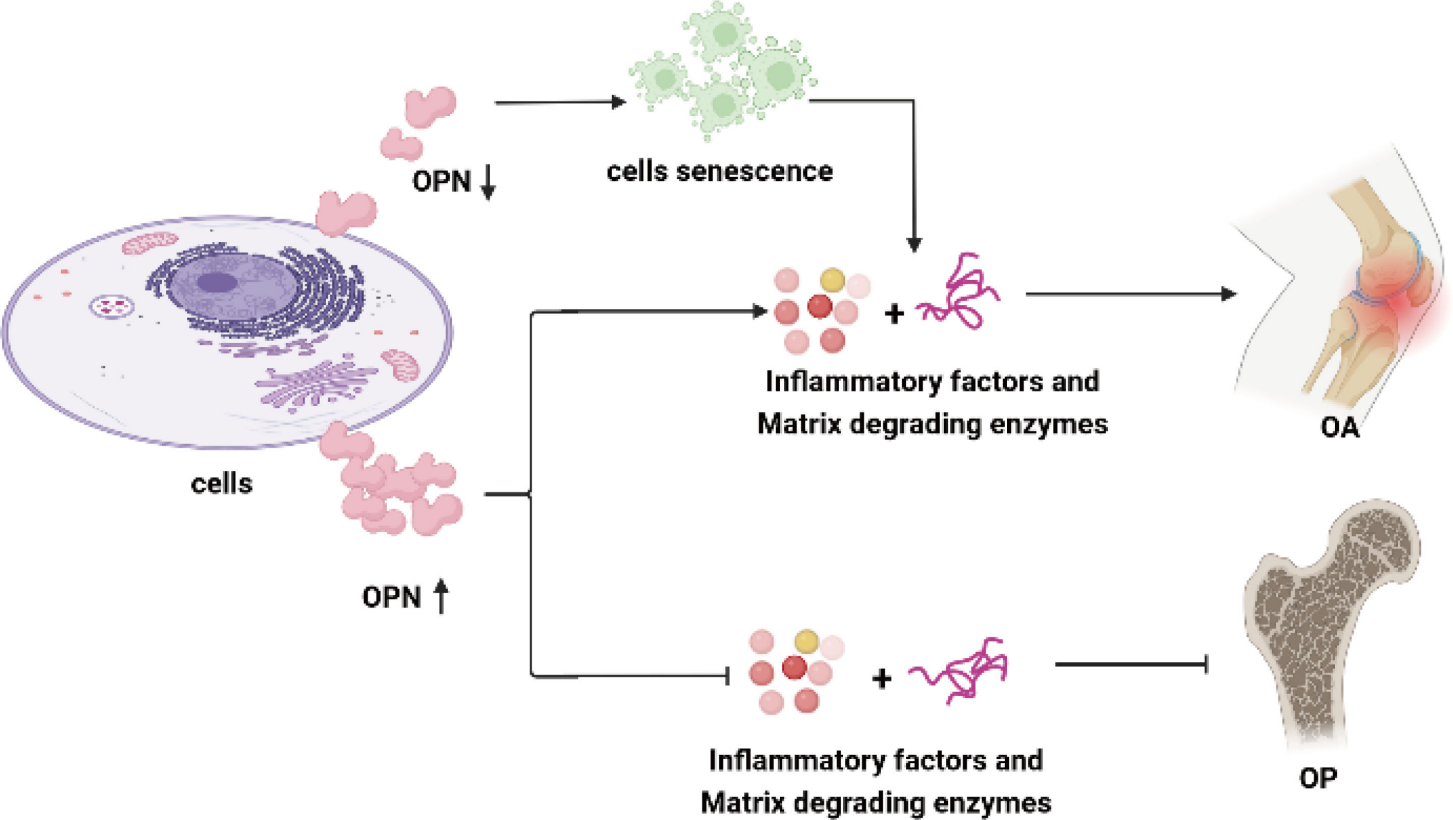
OPN regulates inflammatory activity in OA and OP. OPN plays a controversial role in the inflammation response in OA. Overexpression of OPN in chondrocytes, synoviocytes and subchondral bone stimulates the secretion of proinflammation cytokines and enhances the inflammation activity in OA. OPN-deficiency in chondrocytes leads to chondrocyte senescence and the secretion of cytokines and proteins degraded the articular cartilage. A high level of OPN suppresses the secretion of proinflammation cytokines and matrix-degrading enzymes, alleviates bone mass reduction, and promotes bone formation as well as matrix calcification in OP. The proinflammation cytokines include IL-1β, TNF-ɑ, IL-6, IL-8, NO, iNOS, COX-2, and CRP, while the articular cartilage matrix-degrading enzymes mainly refer to MMP-9, MMP-13, ADAMTS-4, ADAMTS-5 in this review.

Taking together, the above results and studies mainly investigated the role of OPN and its regulating effect on inflammation activity in OA and OP, and further indicated overexpression of OPN or declined OPN levels in chondrocytes, synoviocytes, and osteoblasts could lead to metabolic disturbance in bone diseases as shown in **Figure 1**.

## OPN involves in cartilage and bone metabolism in OA and OP

**Figure 2:**
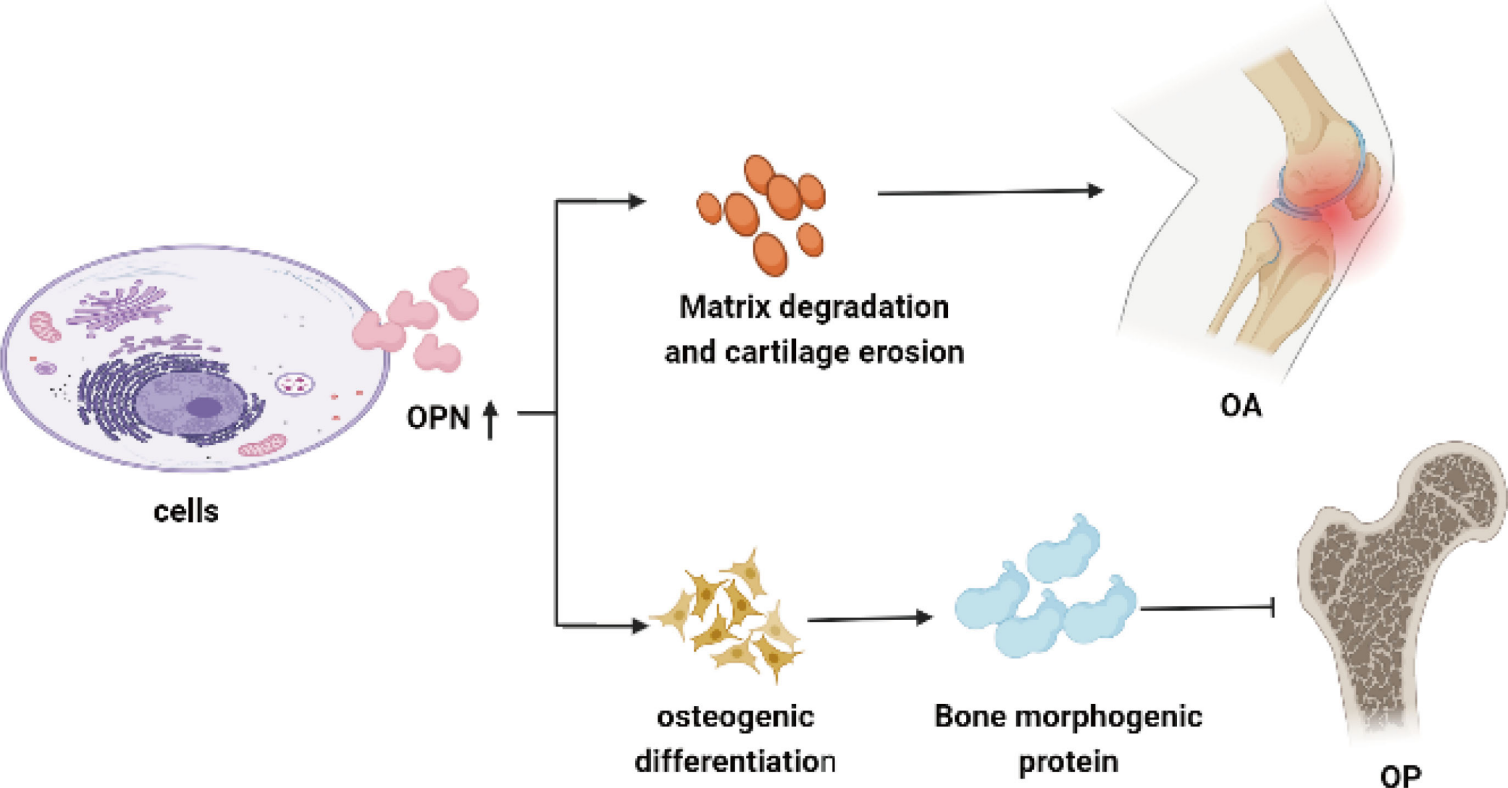
OPN involves in cartilage and bone metabolism in OA and OP. Overexpression of OPN enhances the proliferation of TRAP-positive osteoclasts and secretion of matrix metalloproteinase, leading to bone erosion and cartilage degeneration in OA. High levels of OPN could increase matrix deposition and enhance bone remodeling by promoting osteoblasts differentiation along with the elevated level of bone morphogenetic proteins, resulting in reducing the pathological changes of osteoporosis.

OPN is secreted by many types of cells, such as macrophages, lymphocytes, epithelial cells, vascular smooth muscle cells, chondrocytes, and synovial cells, which exist in a large number of cells and tissues (68–70). It is well known that abnormal expression of OPN in mRNA or protein level is correlated to the onset of OA. Up-regulated OPN and the phosphorylation of OPN in chondrocytes and synovial fluid greatly contribute to and aggravate the severity of OA (10, 19, 68). A previous study shows overexpression of OPN could promote the proliferation of chondrocytes, but suppressed their apoptosis through the NF-κB signaling pathway to regulate the pathological process of OA (70). The elevated OPN serum level is coincident with the tartrate-resistant acid phosphatase (TRAP)-positive osteoclasts and the extent of bone erosion in CIA mice, silencing of OPN using lentiviral-OPN short hairpin RNA presents an opposite result (71). Elmazoglu, et al. (72) found OPN and bone morphogenetic protein-7(BMP-7) were inhibited by S-allylcysteine accompanied by the suppressed inflammation response and decreased IL-1β, IL-6 in chondrocytes which finally alleviate the severity of OA. OPN also mediates subchondral bone remodeling and cartilage degeneration in OA. Animal study shows that higher level of OPN secreted by pre-osteoblasts and osteoblasts in subchondral bone could promote osteoclastogenesis and blood vessels formation in subchondral bone, accelerate the turnover and remodeling of subchondral bone and mediate articular cartilage degeneration induced by subchondral bone metabolism in anterior cruciate ligament transection and destabilization of the medial meniscus (ACLT + DMM) OA model (45). Furthermore, OPN is also on behalf of the osteogenic and chondrogenic differentiation progress, abnormal expression of OPN along with Runx2, Sox9, and OCN may induce metabolic disturbance and cartilage damage in OA (51, 73). Therefore, OPN plays a critical role in regulating the metabolism in chondrocytes, synovial cells, and subchondral bone which greatly influence the physiological process of joint, it should be precise modulated at a proper level for the prevention of onset and aggravation of OA as summarized in **Table 2** and shown **Figure 2**.

**Table 2:** OPN involves bone metabolism in OA and OP.

Reference	disease	Subjects	Test items	Main results
Elmazoglu, et al. (72)	OA	Human OA chondrocyte	BMP	S-allylcysteine exhibits anti-osteoarthritic properties through suppressing the expression of IL-1β, IL-6, OPN and BMP in chondrocytes.
Qian, et al. (71)	OA	Serum and synovial tissue	BMD	Increased serum OPN level in OA and RA is related to the activity of osteoclasts and bone erosion in CIA mice.
Lin, et al. (45)	OA	Subchondral bone and osteoclasts	OCN, Runx2, ALP	OPN promotes osteoclastogenesis in the subchondral bone, regulates OA subchondral bone metabolism and accelerates subchondral bone remodeling.
Cassuto, et al. (53)	OA	OA patients blood samples	BALP, OPG, sclerostin	Total hip arthroplasties patients present the second peak of inflammation five years post-surgery which may stimulate bone remodeling and new synthetic coupling, characterized by increasing bone anabolic transition markers BALP and RANKL/OPG.
Kulsirirat, et al. (73)	OA	Mesenchymal stem cells	Runx2, Sox9, OPN	Andrographolide enhance osteogenesis and chondrogenesis by increasing the expression of osteogenic and chondrogenic differentiation, including Runx2, OPN, Sox9.
Li, et al. (51)	OA	Adipose mesenchymal stem cell	OCN, Runx2	IL-1β treatment inhibits the viability and migration of chondrocytes, enhances cell apoptosis, increase OPN level, decrease the mRNA and protein level of Col1a1, Col2a1, OCN, Runx2. Extracellular vesicles-chitosan oligosaccharide conjugates could reverse the effect of IL-1β on cartilage damage.
Tang, et al. (74)	OP	C57/B6L mice and chondrocytes	Sox9, CyclinD1, PTH1R, Osx, Runx2, ATF4, OCN, OPN	Runx1f/fCol2α1-cre mice exhibits impaired cartilage formation, decreases bone density, accompanied with decreased expression of osteoblast differentiation genes including Osx, Runx2, ATF4 and osteoblast marker genes including OCN and OPN in chondrocytes.
Xiao, et al. (75)	OP	C57BL/6 J mice	Vascular endothelial growth factor (VEGF)	Ovariectomy mice presents osteoporosis of the vertebra and osteochondral remodeling of the endplate with increased endplate porosity and decreased disc volume.
Li, et al. (76)	OP	Human osteoblast-like cells and mice	Collagen I, OCN, OPN	Aucubin increase cortical bone thickness, bone density and tighter trabecular bone in Dex-induced osteoporotic mouse model, up-regulates the expression of collagen I, OCN, OPN in cells.
Gu, et al. (77)	OP	Wistar rats	BMD, BMP2, Runx2, Collagen I, ALP	Anti-Osteoporosis Decoction (AOD) and Yougui Pill treatment effectively inhibits osteoporosis and reduces the broken trabecular bones, increases the level of BMD, ALP levels, accompanied by the increased expressions of BMP2, Runx2, Collagen I and OPN.
Zeng, et al. (78)	OP	Human bone marrow mesenchymal stem cells	Runx2, OCN, and OPN	Artesunate promotes osteoblasts differentiation related proteins Runx2, OCN and OPN expression through activating the Wnt signaling pathway in hBMSCs.
Dong, et al. (79)	OP	Mouse preosteoblast cell lines (MC3T3-E1)	ALP, OCN, OPN, and BMP2	Anagliptin significantly increased matrix deposition and mineralization by osteoblasts, as evidenced by elevated levels of ALP, OCN, OPN, and BMP2.
Lei, et al. (60)	OP	C57B/L6 mice and osteoclasts	Runx2, OPN, TRAP	MLN64 was over-expressed during the process of osteoclast differentiation, up-regulation Runx2 and OPN. The in vivo study suggested that MLN64 deletion reversed streptozotocin-induced trabecular deleterious effects and stimulated bone remodeling.
Yin, et al. (80)	OP	Zebrafish	ALP, hydroxyproline, and TRAP	Evodiamine (EV) can alleviate dexamethasone-induced osteoporosis through increasing the area of bone formation, the content of hydroxyproline and the expression of ALP and TRAP in zebrafish.
Fan, et al. (81)	OP	Sprague-Dawley rats	BMD, ALP, OCN, BMP2, Runx2, TRAP	Myricetin could increase body weight gain and inhibit Dex-induced reduction in BMD, enhanced ALP activity, upregulated OCN, BMP2 and Runx2 levels accompanied with reduced TRAP activity and CTX level.
Wu, et al. (82)	OP	Human plasma samples	B lymphocyte chemoattractant (BLC) and BMP	Inflammatory cytokines were closely related with OP, BLC, OPN and IGFBP4 are greatly related to the pathogenesis of OP.
Lee, et al. (83)	OP	Female ICR mice	collagen type I, BMP2 and OPN	Osteo-F ameliorated bone loss by increasing bone forming molecules including BMP-2 and OPN in osteoporosis.
Marycz, et al. (84)	OP	MC3T3-E1cell lines	OPN, OCN and ALP	PRHD@MnFe2O4 protect osteoporosis through enhancing the expression of bone remodeling markers including OPN, osteocalcin (OCL) and ALP in pre-osteoblasts.
Shao, et al. (85)	OP	MC3T3-E1 cells	ALP, osteocalcin, OPN and BMP-2	Trelagliptin increased the activity of ALP and promoted osteoblastic calcium deposition. Additionally, Trelagliptin upregulated ALP, osteocalcin, OPN, and BMP2 and Runx2 in MC3T3-E1 cells.

Osteoporosis is a systemic skeletal disorder characterized by systemic damage to bone mass and microstructure, which is caused by bone metabolism disorders and is the main reason for fragility fractures in aged people (30, 86, 87). Notably, OPN plays an important role in bone strength and bone remodeling. The role of OPN involved in cartilage and bone metabolism of OP has been greatly investigated in many previous studies and the effect of OPN levels on osteoporosis is gaining more and more attention, especially those OPN exits in serum, chondrocytes, osteoclasts, and osteoblasts.

A large number of previous studies through animal experiments have shown that OPN has a protective effect on osteoporosis and OPN-deficient mice by oophorectomy are resistant to osteoporosis (88–90). Chondrocyte-specific genes knockout mice also showed impaired cartilage formation, decreased bone density and an osteoporotic phenotype with the decreased osteoblast marker genes including OPN and OCN in osteoblasts, as well as the expression of osteoblast differentiation regulation genes such as Osx, Runx2 and ATF4 (74). Ovariectomy mice presented an osteoporosis phenotype mainly on osteochondral remodeling accompanied by increasing the endplate porosity and decreasing the bone volume, the changes of OPN, OCN, Osx in osteoblast and serum which influence the osteoblast differentiation is responsible for this pathological process (75, 76). Clinical studies have found that serum level of OPN, as biomarkers for early diagnosis of osteoporosis in postmenopausal women, is positively related to the severity of osteoporosis. The postmenopausal women have a higher OPN serum level compared to those premenopausal women, meanwhile the higher OPN level shows a negatively correlated relationship with the weight, height, and BMD (31, 32, 35). Previous scholars found the serum OPN level in the menopausal females was 15.4 (ng/ml) compared to non-menopausal females with a lower OPN level of 7.8 (ng/ml), moreover, the higher OPN level also indicated an approximately 2.97-fold risk of osteoporosis compared with the persons with low serum OPN levels (12). Vancea et al. (33–35) also confirmed the negatively relationships between OPN and BMD in the postmenopausal women, they considered postmenopausal women exhibited higher serum OPN levels may have a lower BMD and higher risk of OP than the premenopausal women. Wu et al. (82) adopted an extreme sampling design to systemically screen the OP-related cytokines in serum of postmenopausal women, further verified the OPN and BLC modulate the bone metabolism by inhibiting bone formation and promoting bone resorption. Since OPN has a major effect on osteoclasts through regulating the bone metabolism and bone remodeling, plenty of scholars have devoted great energy to investigating the role of OPN in OP and found new drugs targeting OPN to treat osteoporosis. Dong et al. (79) investigated the effects of anagliptin on the differentiation and mineralization of osteoblasts and discovered that anagliptin significantly increased matrix deposition and mineralization by increasing the activity of osteoblasts as evidenced by elevated levels of ALP, OCN, OPN, and BMP-2. Other studies also confirmed increased expression of OPN in mRNA or protein level of osteoclasts protects the osteoporosis to promote osteogenic differentiation and inhibits osteoclastogenesis. The potential therapeutic effects of elevated OPN activity on osteoporosis mainly reflected on increasing body weight gain and BMD, with the assistance effect of up-regulated level of ALP, OCN, BMP-2 and reduced tartrate-resistant acid phosphatase (TRAP) activity as well as C-terminal telopeptide of type I collagen (CTX-I) level (81, 85, 91). In addition, some scholars also found traditional Chinese medicine tonifying prescriptions have potential therapeutic effects on osteoporosis in ovariectomy-induced rat model. The traditional Chinese medicine mainly targeted to increase the expression of OPN, BMP-2 and Runx2 which finally resulted in reducing the broken trabecular bones in femur bones and increasing the activity of ALP combined with enhanced the content of total bone mineral density in osteoporosis rats (77). Due to the important biological effect of OPN on osteoporosis, its expression and physiological effect in mesenchymal stem cells (MSC) has been greatly studied. Recently, researchers confirmed that the therapeutical effect of MSCs on osteoporosis relied on the elevated extracellular Ca^2+^ promoted cell proliferation and matrix mineralization of MSCs. Moreover, the enhancement of MSCs under up-regulated extracellular Ca^2+^ condition is induced by the elevated OPN level (92). It is further found OPN not only significantly promoted the proliferation of MSCs, but also activated the migration and regulate the cell stiffness through integrin and the Wnt signaling pathway which accelerates the osteoblasts differentiation of MSCs indicated by overexpression of osteoblasts differentiation related proteins (78, 93, 94).

## Pharmaceutical interventions of OPN in OA and OP

The complex effect of OPN means that regulating its expression is a complex and challenging process. Recently, some new and classic drugs have investigated the effect of OPN on OA and OP. Pharmaceutical interventions of OPN in OA have hitherto mainly focused on the OPN level in chondrocytes, synovial tissue and synoviocytes. Elmazoglu et al. (72) found that S-allylcysteine could inhibit the IL-1β, IL-6 and OPN in chondrocytes by suppressing the activity of the p-JNK/pan-JNK signaling pathway. The inhibition of OPN and these cytokines finally lead to the up-regulated level of peroxidase and type-II-collagen to delay the progress of OA. Li et al. (51) investigated the effect of chitosan oligosaccharides which are packed into the extracellular vesicles in OA. They verified that chitosan oligosaccharides could reverse IL-1β induced chondrocytes apoptosis and the inhibition of viability and migration of chondrocytes, furthermore, they also discovered the therapeutical effect of chitosan oligosaccharides on cartilage damage was by down-regulating the level IL-1β, OPN, and p53 accompanied by the increased level of Col2a1, OCN, and Runx2 *via* PI3K-Akt pathway. Isorhamnetin also reduced MIA-induced knee swelling by significant reduction of articular cartilage damage through regulating the OPN level in rats. The protective effect of isorhamnetin on OA was through the inhibition of OPN, NO, PGE2, iNOS and COX-2 (52). Slovacek et al. (49) showed that OPN and MMP-9 levels were significantly elevated in OA, the increased level of OPN and MMP-9 is stimulated by inflammatory markers, such as IL-1, IL-6, and CRP. Previous researchers found adiponectin aggravates bone erosion by promoting OPN production in synovial tissue while silencing of OPN with lentiviral-OPN short hairpin RNA could reduce the number of TRAP-positive osteoclasts and the extent of bone erosion in CIA mice (71). In addition, the microRNA(miR) could also influence the expression of OPN in OA. MiR-181c could greatly inhibit the effect of OPN on stimulating the secretion of MMP-13, IL-6, and IL-8 along with repressing synoviocyte proliferation which finally alleviated local inflammation activity and cartilage damage in joints, severed as a therapeutic strategy for OA (50).

The multiple effects of OPN on different types of cells indicates that modulation of OPN in OP represents a definite challenge. Our current understanding of pharmaceutical interventions of OPN in OP is discussed in the following sections and summarized in **Table 3**. Gao et al. (58) found salvianolic treatment ameliorated osteopenia and improved bone quality in rats, the potential mechanism may rely on increasing Osx, OPN, and Col1a1 levels in TNF-α-induced MC3T3-E1 osteoblasts through regulating the RANKL/RANK/OPG signaling pathway. Monotropein was also found to play an important role in regulating the OPN metabolism in MC3T3-E1 cells. Research indicated that monocropping was able to increase the proliferation and activity of ALP, bone matrix mineralization and the expression of bone matrix protein OPN accompanied by decreasing the production of IL-6 and IL-1β *via* suppressing the activity of the NF-κB signaling pathway (61). Choi et al. (96) found that Palmul-tang could also increase bone mineral content and improve bone mineral density to treat OP. Furthermore, they indicated that the therapeutic effect of Palmul-tang mainly depended on up-regulating the expressions of BMP-2, Runx2, and Osx with its downstream factors ALP and OPN through the BMP-2 signaling pathway. Increased level of OPN also influenced the osteoblasts differentiation, matrix deposition and mineralization along with the high level of ALP, OCN, OPN, and BMP-2 which was related to the Wnt/β-catenin signaling pathway with the interventions of anagliptin and artesunate (78, 79). Apart from the above biomarkers such as BMP-2, Runx2, Osx and ALP, the serum OPN level was also negatively correlated with bone turnover markers mainly including parathyroid hormone, lumbar spine BMD, femoral neck BMD and positively associated with type I procollagen amino-terminal propeptide (PINP), carboxy-terminal cross-linking telopeptide of type I collagen (CTX) in the OP patients (35). Furthermore, they indicated CTX was independent predictor of serum OPN while vitamin D was not correlated to OPN in adults (32, 97). The animal model showed that up-regulated OPN could enhance the activity of ALP, OCN, and BMP-2 in dexamethasone-induced OP, the anti-osteoporosis function of myricetin *in vivo* may be due to the promoting osteogenic differentiation and matrix mineralization effect caused by OPN (83).

**Table 3:** Drug therapies for treatment of OA and OP.

Reference	Disease	Drugs	Subjects	Signaling pathways	Main results
Qiyuan Wang, et al. (50)	OA	miR-181c	Synoviocytes	/	MiR-181c significantly repressed synoviocyte proliferation, as well as the levels of OPN, MMP13, IL-6, and IL-8.
Shenglong Li, et al. (51)	OA	Chitosan oligosaccharides	Chondrocytes	/	Chitosan oligosaccharides could reverse the effect of IL-1β on inhibiting chondrocytes viability and migration, moreover, chitosan oligosaccharides could reverse the upregulated expression of IL-1β, OPN, and p53 induced by cartilage injury.
Tsai Sen-Wei, et al. (52)	OA	Isorhamnetin	Sprague-Dawley rats	/	Isorhamnetin may reduce MIA-induced knee swelling by significantly reduction of articular cartilage damage in rats. The protective effect of isorhamnetin on OA was through the inhibition of OPN, NO, PGE2, iNOS and COX-2.
Zubeyir Elmazoglu, et al. (72)	OA	S-allylcysteine	Human OA chondrocyte	p-JNK/pan-JNK signaling pathway	S-allylcysteine (SAC) inhibited reactive oxygen species (ROS), lipid hydroperoxides (LPO), IL-1β, IL-6, OPN and increased glutathione peroxidase (GPx) and type-II-collagen to control the progression of OA.
Qian, et al. (71)	OA	Adiponectin	Serum and synovial tissue	/	Adiponectin aggravates bone erosion by promoting OPN production in synovial tissue of rheumatoid arthritis.
Kulsirirat, et al. (73)	OA	Andrographolide	Mesenchymal stem cells	/	Andrographolide could upregulate the expression of genes related to osteogenic and chondrogenic differentiation, including *Runx2*, *OPN*, *Sox9*, and *Aggrecan* in mesenchymal stem cells.
Marycz, et al. (57)	OP	NHAp/IO@miR-21/124	MC3T3-E1 cells	/	NHAp/IO@miR-21/124 could enhance osteogenesis through increasing osteogenic markers Runx-2, OPN, Coll-1 level.
Xiang Gao, et al. (58)	OP	Salvianolate	Lewis rats	RANKL/RANK/OPG signaling pathway	Salvianolate treatment ameliorate osteopenia and improved bone quality through increasing the Osterix, OPN, Runx2 level and decrease TNF-α, IL-6 level in serum.
Yu-Qiong He, et al. (61)	OP	Monotropein	C57/BL6 mice and MC3T3-E1 cells	NF-κB signaling pathway	Monotropein inhibited bone mass reduction and improved bone micro-architectures by enhancing bone formation and blocking increased secretion of inflammatory cytokines in osteoporotic mice.
Miranda, et al. 959)	OP	strontium ranelate	Wistar rats	NF-κB signaling pathway	Strontium ranelate-treated groups presented higher staining of OPN and BSP which was beneficial to bone healing and the expression of bone markers.
La Yoon Choi, et al. (96)	OP	Palmul-tang	Female ICR mice	BMP-2 signaling pathway	Palmul-tang replenished bone marrow cavity and increased collagen deposition in bone marrow cells of femur, furthermore, restored the bone minerals and improvement of bone integrity through increasing the expressions of BMP-2, Runx2 and OSX with its downstream factors, ALP, OPN and BSP-1.
Li, et al. (76)	OP	Aucubin	MG63 cells	/	Aucubin increase cortical bone thickness, bone density and tighter trabecular bone in Dex-induced osteoporotic mouse model through increasing the level of collagen I, OCN, OPN in cells.
Zeng, et al. (78)	OP	Artesunate	hBMSCs	Wnt signaling pathway	Artesunate increased the osteoblasts differentiation related protein expression through activating the Wnt signaling pathway.
Dong, et al. (79)	OP	Anagliptin	MC3T3-E1 cells	Wnt/β-catenin signaling pathway	Anagliptin increased matrix deposition and mineralization by increased the expression of ALP, OCN, OPN, and BMP-2.
Yin, et al. (80)	OP	Evodiamine	Zebrafish	MMP3-OPN-MAPK pathway	Evodiamine increased the area of bone formation through MMP3-OPN-MAPK pathway in zebrafish.
Fan, et al. (81)	OP	Myricetin	Sprague-Dawley rats	/	Myricetin promoted osteoblast differentiation and mineralization, accompanied by increases in BMP2, Runx2, ALP, OCN, Col1a and OPN levels.
Lee, et al. (83)	OP	Osteo-F	Female ICR mice	/	Osteo-F-treatment increased the Bone mineral content and BMD through up-regulating the mRNA expressions of collagen type I, BMP-2 and OPN in ovariectomized mice.
Huang, et al. (91)	OP	Psoralen	hBMSCs	TGF-β/Smad3 signaling pathway	Psoralen accelerates osteogenic differentiation by increasing the expression of BMP4, OPN, Osterix, Runx2, TGF-β1, TGF-β RI and p-Smad3 through activating the TGF-β/Smad3 pathway.
Marycz, et al. (84)	OP	PRHD@MnFe2O4	MC3T3-E1 cells	/	PRHD@MnFe2O4 could be a potential agent in osteoporosis treatment through enhancing expression of bone remodeling genes mainly including OPN, OCL and ALP.

Taking together, the pharmaceutical interventions of OPN in bone metabolism present different biological effects due to the multiple types of cells. Generally speaking, the current drug treatment mainly depended on reducing the OPN level in chondrocytes and synovial cells to suppress inflammatory activity alleviate cartilage erosion, and delay the course of OA. While it referred to OP, the pharmaceutical effect of drug intervention on OPN is to increase the expression level of OPN in osteoclasts and stem cells. It is contrary to the study of drug intervention in OA, the increased OPN level, as well as its related cytokines BMP-2, OCN, and Runx2, could promote bone formation and enhance bone mineral density to achieve the purpose of treating OP.

## Conclusion

In recent years, there has been growing interest in trying to identify the physiological effect of OPN on skeleton diseases. OPN acts as a secreted protein that participates in the progress of bone remodeling and bone metabolism which are relevant to many bone metabolism disturbance diseases. OPN regulates the inflammation activity both in OA and OP, and plays controversial roles in the inflammation response for either overexpression of OPN level in chondrocytes and synoviocytes or OPN-deficiency in cells residents in joints may stimulate and enhance the inflammation activity in OA as the **Figure 1** shown. While the higher level of OPN could suppress the secretion of proinflammation cytokines and inflammation activity, alleviate bone mass reduction, and promote bone formation as well as matrix calcification in OP.

As referred to as the role of cartilage and bone metabolism, OPN is a biological marker of OA severity. Overexpression of OPN enhances the proliferation of chondrocytes and TRAP-positive osteoclasts along with the osteogenic and chondrogenic differentiation progress, which finally leads to bone erosion and cartilage degeneration in OA. High levels of OPN could increase matrix deposition and enhance bone remodeling by promoting osteoblasts differentiation along with the elevated level of ALP, OCN, and BMP-2 which finally resulted in reducing the pain and pathological changes of osteoporosis. Low activity of OPN could increase fracture sensitivity in patients with osteoporosis, especially for those postmenopausal women as **Figure 2** shown. Due to the above physiological role of OPN in OA and OP, we do deem that OA and OP influenced each other for the reason that OA is associated with bone formation as seen in subchondral sclerosis and osteophyte formation, and the tendency to accumulate bone in the subchondral area could increase the onset of OA. In contrast, once OA is established, the pain and reduced mobility reduce bone mass, particularly in the affected limb. The pain and loss of joint function in OA patients could result in muscle loss and postural instability, which subsequently increased fracture risk. OPN may serve as a bridge role in regulating bone metabolism and signal transduction between the disease of osteoarthritis and osteoporosis, and it should be precisely modulated at a proper level for either higher OPN level or lower OPN activity may induce to disrupt the balance of bone tissue. However, there are still many details and effects of OPN on bone metabolism and bone-related disease not been elucidated. Further in-depth studies on the physiological function of OPN provide new ideas and directions for exploring and clarifying the pathogenesis of bone metabolic diseases, targeting OPN may provide new clinical therapeutic orientations and values for OA and OP.

## Author contributions

All the authors researched the data for the article, provided substantial contributions to discussions of its content, wrote the article and reviewed and/or edited the manuscript before submission. All authors contributed to the article and approved the submitted version.

## Funding

This work was supported by the National Key R&D Program of China (No.2019YFA0111900), the National Natural Science Foundation of China (No.81501923 and No. 82072506), Provincial Natural Science Foundation of Hunan (No.2020JJ3060), Administration of Traditional Chinese Medicine of Hunan Province (No.2021075), InnovationDriven Project of Central South University (No.2020CX045), Wu Jieping Medical Foundation (No.320.6750.2020-03-14), Rui E (Ruiyi) Emergency Medical Research Special Funding Project (No.R2019007), and Independent Exploration and Innovation Project for Postgraduate Students of Central South University (No.2021zzts1037).

## Conflict of interest

The authors declare that the research was conducted in the absence of any commercial or financial relationships that could be construed as a potential conflict of interest.

## Publisher’s note

All claims expressed in this article are solely those of the authors and do not necessarily represent those of their affiliated organizations, or those of the publisher, the editors and the reviewers. Any product that may be evaluated in this article, or claim that may be made by its manufacturer, is not guaranteed or endorsed by the publisher.

## Glossary

**Table:**

OA	osteoarthritis
OP	osteoporosis
OPN	osteopontin
SMC	smooth muscle cells
SIBLING	small integrin-binding ligand N-linked glycoprotein
Eta-1	early T cell activa-tion gene-1
BMD	bone mineral density
CRP	C-reaction protein
ACLT	anterior cruciate ligament transection
DMM	destabilization of the medial meniscus
MMP-9	matrix metalloproteinase 9
OCN	osteocalcin
IMO	inflammation mediated osteoporosis
COX-2	cyclooxygenase-2
PGE2	prostaglandin E2
TRAP	tartrate-resistant acid phosphatase
BMC	bone mineral content
CIA	collagen-induced arthritis
ALP	alkaline phosphatase
CTX-I	C-terminal telopeptide of type I collagen
BMP-7	bone morphogenetic protein-7
IL	interleukin
TNF-α	tumor necrosis factor alpha
MSC	mesenchymal stem cells
NF-kB	nuclear factor kappa-B
iNOS	inducible nitric-oxide synthase
LPS	Lipopolysaccharide
ADAMTS	a disintegrin and metalloproteinase with thrombospondin motifs
Col2a1	type II collagen alpha 1 chain
Sox9	SRY-box transcription factor 9
Acan	Aggrecan
Osx	Osterix
HFD	high-fat diet
ECM	extracellular matrix
HA	hyaluronan
miR	microRNA
